# Profiling of Patients with COPD for Adequate Referral to Exercise-Based Care: The Dutch Model

**DOI:** 10.1007/s40279-020-01286-9

**Published:** 2020-04-24

**Authors:** Martijn A. Spruit, Alex Van’t Hul, Hilde L. Vreeken, Emmylou Beekman, Maria H. T. Post, Guus A. Meerhoff, Anne-Loes Van der Valk, Cor Zagers, Maurice J. H. Sillen, Martijn Vooijs, Jan Custers, Jean Muris, Daniel Langer, Jos Donkers, Marleen Bregman, Leendert Tissink, Erik Bergkamp, Johan Wempe, Sarah Houben-Wilke, Ingrid M. L. Augustin, Eline Bij de Vaate, Frits F. M. Franssen, Dirk Van Ranst, Hester Van der Vaart, Jeanine Antons, Mitchell Van Doormaal, Eleonore H. Koolen, Philip Van der Wees, Renée Van Snippenburg, Daisy J. A. Janssen, Sami Simons

**Affiliations:** 1 Department of Research and Development, CIRO+, Horn, The Netherlands; 2 Department of Respiratory Medicine, Maastricht University Medical Center (MUMC+), Maastricht, The Netherlands; 3 NUTRIM School of Nutrition and Translational Research in Metabolism, Maastricht, The Netherlands; 4 REVAL Rehabilitation Research Center, BIOMED Biomedical Research Institute, Faculty of Rehabilitation Sciences, Hasselt University, Diepenbeek, Belgium; 5 Department of Pulmonary Diseases, Radboud University Medical Center, Nijmegen, The Netherlands; 6 Royal Dutch Society for Physical Therapy (KNGF), Amersfoort, The Netherlands; 7 Research Centre for Autonomy and Participation of Persons With a Chronic Illness, Zuyd University of Applied Sciences, Heerlen, The Netherlands; 8 CAPHRI School for Public Health and Primary Care, Maastricht University Medical Centre, Maastricht, The Netherlands; 9 ParaMedisch Centrum Zuid, Physical Therapy Section in Multidisciplinary Centre, Sittard, The Netherlands; 10 Association of Exercise Therapy Cesar and Mensendieck (VvOCM), Utrecht, The Netherlands; 11 FysioCompany Van Mourik & Van Der Valk, ‘s-Hertogenbosch, The Netherlands; 12 Fysiotherapiecentrum Lombok, Utrecht, The Netherlands; 13 Department of Pulmonary Rehabilitation, Basalt Rehabilitation, Leiden, The Netherlands; 14 Institute of Human Movement Studies, University of Applied Science, Utrecht, The Netherlands; 15 Department of Family Medicine, Care and Public Health Research Institute (CAPHRI), Maastricht, The Netherlands; 16 Department of Rehabilitation Sciences, Faculty of Kinesiology and Rehabilitation Sciences, Research Group for Rehabilitation in Internal Disorders, Leuven, KU Belgium; 17 Horn, The Netherlands; 18 Department of Physiotherapy, Merem Medische Revalidatie, Hilversum, The Netherlands; 19 FysioHolland-Jofib, Oud Gastel, The Netherlands; 20 Department of Physiotherapy, Spaarne Gasthuis, Haarlem, The Netherlands; 21 Center for Rahabilitation, University Medical Center Groningen, University of Groningen, Groningen, The Netherlands; 22 Merem Medische Revalidatie, Hilversum, The Netherlands; 23 Revant, Breda, The Netherlands; 24 Department of Pulmonary Diseases and Tuberculosis, University Medical Center Groningen, University of Groningen, Groningen, The Netherlands; 25 Department of Pulmonary Diseases, Radboudumc Dekkerswald, Nijmegen, The Netherlands; 26 Department of Rehabilitation and IQ Healthcare, Radboudumc, Radboud Institute for Health Sciences, Nijmegen, The Netherlands; 27 Saltro Diagnostic Center, Utrecht, The Netherlands; 28 Department of Health Services Research, CAPHRI School for Public Health and Primary Care, Faculty of Health Medicine and Life Sciences, Maastricht University, Maastricht, The Netherlands

## Abstract

A loss of physical functioning (i.e., a low physical capacity and/or a low physical activity) is a common feature in patients with chronic obstructive pulmonary disease (COPD). To date, the primary care physiotherapy and specialized pulmonary rehabilitation are clearly underused, and limited to patients with a moderate to very severe degree of airflow limitation (GOLD stage 2 or higher). However, improved referral rates are a necessity to lower the burden for patients with COPD and for society. Therefore, a multidisciplinary group of healthcare professionals and scientists proposes a new model for referral of patients with COPD to the right type of exercise-based care, irrespective of the degree of airflow limitation. Indeed, disease instability (recent hospitalization, yes/no), the burden of disease (no/low, mild/moderate or high), physical capacity (low or preserved) and physical activity (low or preserved) need to be used to allocate patients to one of the six distinct patient profiles. Patients with profile 1 or 2 will not be referred for physiotherapy; patients with profiles 3–5 will be referred for primary care physiotherapy; and patients with profile 6 will be referred for screening for specialized pulmonary rehabilitation. The proposed Dutch model has the intention to get the right patient with COPD allocated to the right type of exercise-based care and at the right moment.

## Key Points

**Table:** 

To date, use of primary care physiotherapy or specialized pulmonary rehabilitation programs is very limited in patients with COPD (5.0 and 0.2%, respectively), while a larger proportion of these patients clearly qualify for this type of care.
The current organization of Dutch healthcare needs to make a transition towards an adequate referral of patients with COPD to the different types of exercise-based care, including a healthy lifestyle advise, physiotherapy and/or specialized pulmonary rehabilitation programs.
Disease stability, disease burden, physical capacity and physical activity are important traits to get the right patient allocated to the right type of exercise-related care and at the right moment, irrespective of the degree of airflow limitation.

## Introduction

Despite medical treatment by the general practitioner, an impaired physical, emotional and/or social functioning has been frequently reported in Dutch primary care patients with chronic obstructive pulmonary disease (COPD) [1–3]. These abnormalities can co-occur in different combinations, regardless of the degree of airflow limitation. Similar findings have been reported in patients with COPD who were under the care of Dutch pulmonologists [4, 5].

A loss of physical functioning (i.e., a low physical capacity and/or a low physical activity) is a common feature in patients with COPD [3, 6, 7]. Exercise-based interventions (combined with self-management education) and/or physical activity coaching programs can result in clinically relevant improvements in daily symptom burden, physical capacity, physical activity and health status compared to usual care in patients with COPD [8]. An interdisciplinary comprehensive pulmonary rehabilitation (PR) program is defined as ‘*a comprehensive intervention based on a thorough patient assessment followed by patient-tailored therapies, which include, but are not limited to, exercise training, education, and behavior change, designed to improve the physical and psychological condition of people with chronic respiratory disease and to promote the long-term adherence of health-enhancing behaviors*’ [9]. Such programs have shown to also improve the performance of activities of daily living, to increase self-efficacy, and to lower the degree of care dependency and healthcare utilization [5, 10–14] in COPD patients with a combination of physical, emotional and/or social treatable traits. Even though safety and efficacy of these interventions are clear, referral by physicians remains poor [15].

## Current Clinical Practice

Current guidelines state that the degree of clinical complexity should determine the types of (pharmacological and non-pharmacological) interventions provided, ranging from healthy lifestyle advise combined with recommended drug therapy [16, 17], up to a comprehensive, inpatient, interdisciplinary PR program for patients with multiple physical, emotional and/or social treatable traits at the time of referral [10, 18].

Since January 2019, patients with COPD in the Netherlands are entitled to reimbursement of the costs of physiotherapy (or exercise therapy Cesar/Mensendieck) provided in the primary care setting via the basic national healthcare insurance [19]. Indeed, the National Healthcare Institute advised the Dutch Minister of Health, Welfare and Sport that patients with moderate to very severe COPD (FEV1 < 80% predicted) are eligible to receive a maximum number of reimbursed physiotherapy sessions in the first 12 months of treatment and during the enduring maintenance phase. However, GOLD C/D patients who suffer from a new exacerbation during/after treatment are not entitled to reimbursement of the costs of extra physiotherapy sessions. Moreover, the maximum number of sessions is solely based on the GOLD ABCD classification at the time of referral, and excludes GOLD 1 patients (Table 1). However, a subgroup of GOLD 1 patients can suffer from an impaired physical functioning [3], which justifies early referral to exercise-related care, such as physiotherapy [20]. Also respiratory symptom burden and exacerbation history (the two attributes required to classify patients into GOLD ABCD) have not been validated to identify the right candidates for the right type of exercise-based care in patients with COPD. Consequently, highly-symptomatic GOLD B patients are currently entitled to a lower maximum number of reimbursed physiotherapy sessions than the no/low-symptomatic GOLD C patients (Table 1). Moreover, potentially important targets for physiotherapy, such as exercise intolerance and physical inactivity, are ignored in current Dutch referral rules, but vary to a great extent in the different GOLD groups and cannot be derived truthfully from the total scores of the Clinical COPD Questionnaire (CCQ) or COPD Assessment Test (CAT), the questionnaires that are used to qualify patients as GOLD A/C or B/D [21]. Finally, a large variation in treatable traits is present within specific GOLD groups [22]. This necessitates additional assessment of the physical, emotional and social status of patients with COPD to truly understand the disease burden, and to put together a patient-tailored treatment program, including exercise-based care.

**Table 1:** The number of reimbursed physiotherapy sessions in primary care for patients with COPD (since January 2019)

	GOLD group A^a^	GOLD group B^a^	GOLD group C^a^	GOLD group D^a^
Number of session in the 1st year	5	27	70	70
Number of sessions in the enduring maintenance phase	0	3	52	52

^a^Forced expiratory volume in the first second (FEV1) < 80% predicted

## The 2020 Dutch Model

**Fig. 1:**
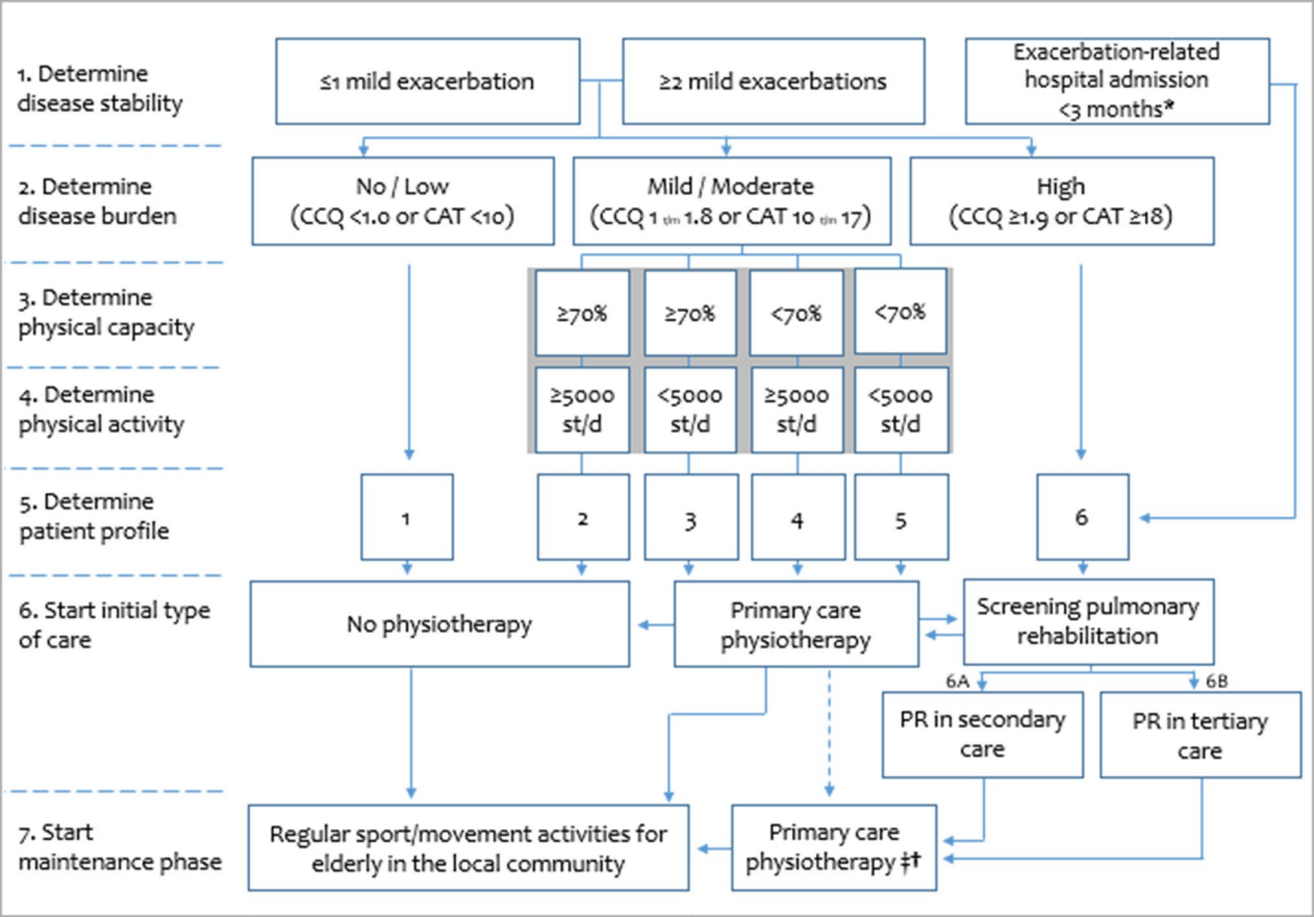
Flowchart for exercise-based care for patients with COPD. *CCQ* Clinical COPD Questionnaire, *CAT* COPD Assessment Test, *%* % predicted value, *st/d* steps per day. *During hospital admission patients with COPD should be offered exercise-based physiotherapy in addition to regular respiratory physiotherapy [58]. ^‡^Frail patients with COPD in the palliative phase of the disease, those who are on the waiting list for lung transplantation, those who are on long-term oxygen therapy, those who are on non-invasive ventilation, and/or those with comorbidities which seriously affect physical capacity/activity. ^†^Patients who are willing to pay out of pocket. Gray area: two 1-h pre-treatment screening sessions to do an intake, and to assess physical capacity and physical activity (as described in the text). Patients who are do not enter a pulmonary rehabilitation program after the screening, will be referred for exercise-based primary care, according to the described profiling 2–5

An ad hoc Task Force, including experts in the field of physiotherapy, exercise therapy (Cesar and Mensendieck), rehabilitation sciences, respiratory medicine, general medicine, elderly care medicine and patient representatives, put together an alternative practice- and experience-based proposal (Fig. 1). This newly proposed flowchart includes an initial patient profiling at the office of the general practitioner or pulmonologist, using a short and simple questionnaire (CCQ or CAT, which both go beyond respiratory symptoms [23, 24]) to determine the degree of disease burden [24, 25]. The modified Medical Research Council (mMRC) dyspnoea scale is *not* proposed as initial screening tool, as its focus is too limited to truthfully capture the multidimensional symptoms/limitations of patients with COPD [23, 27].

### No-to-Low Disease Burden

Patients with no-to-low self-reported disease burden (CCQ < 1.0 point; CAT < 10 points [26]) will most probably have no clearly defined exercise-related treatment goal for which they need advice/supervision from a healthcare professional. Therefore, the general practitioner will provide these patients with healthy lifestyle advice, including an advice to remain regularly physically active [17]. However, there will be no referral for additional physiotherapy or PR (Fig. 1; patient profile 1). Once yearly or if a physician-treated exacerbation occurs earlier, the patient profiling re-starts from the top of Fig. 1.

### Mild-to-Moderate Disease Burden

Patients with a mild-to-moderate self-reported burden of disease (CCQ 1.0–1.8 points; CAT 10–17 points [24]) will be referred to a physiotherapist (or exercise therapist Cesar/Mensendieck; in the primary or secondary care setting, with sufficient knowledge and skills in exercise-based care of patients with chronic lung diseases) to assess physical functioning during two screening visits. During the initial screening visit, patients will undergo an intake by the physiotherapist and a field-based exercise test, such as the 6-min walk test (6MWT) [28, 29]. Moreover, the patients will also receive a step counter/accelerometer, which will be returned at the second screening visit, 1 week later. During this second screening visit physical activity is evaluated and the 6MWT is repeated [30]. Patients with a rather preserved physical capacity (> 70% predicted value [3]) and physical activity (> 5000 steps per day [3]) will receive healthy lifestyle advice, but no additional professional allied healthcare (Fig. 1; patient profile 2). Once yearly or if a physician-treated exacerbation occurs earlier, the profiling re-starts from the top of Fig. 1. Patients with profiles 1 or 2 seem to be good candidates to participate in regular sports/walking activities as organized for elderly in the local communities, which stimulates regular walking in patients with COPD and their loved ones [31].

**Fig. 2:**
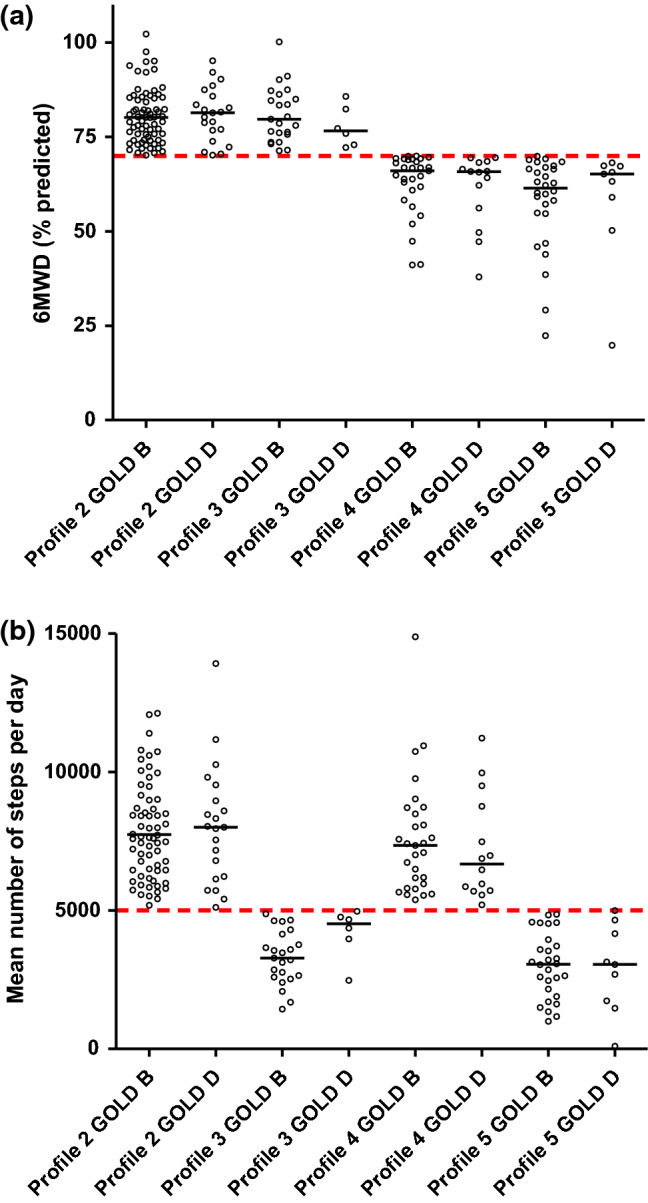
**a** Physical capacity in patients with COPD after stratification for patient profile (2–5) and GOLD group (B or D). **b** Physical activity in patients with COPD after stratification for patient profile (2–5) and GOLD group (B or D). *6MWD* 6 min walk distance, *GOLD* Global initiative for chronic Obstructive Lung Disease. Data are derived from a secondary analysis of the data of Koolen et al. [3]

For patients with a mild-to-moderate self-reported disease burden, accompanied by an impaired physical function [low physical capacity (≤ 70% predicted value) and/or low physical activity (≤ 5000 steps per day)], intervention will start in the primary care physiotherapy setting (Fig. 1; patient profiles 3–5). In profile 3, the treatment will focus on physical activity coaching to increase the daily physical activity [32]; in profile 4, the treatment will focus on exercise training to increase the physical capacity [33, 34]; and in profile 5 it will be a combination thereof. As profile 5 patients have to increase their physical capacity and physical activity, it seems fair that they are entitled to the reimbursement of the costs of more physiotherapy sessions provided in the primary care setting compared to profile 3 or 4 patients (Table 2). The current reimbursement of primary care physiotherapy sessions for the GOLD D patients (Table 1) will suffice for the newly proposed model. In contrast, the number of reimbursed physiotherapy sessions for patients with COPD GOLD B needs to increase to enable the proposed model. It is hard to understand why GOLD D patients are entitled to a higher number of primary care physiotherapy sessions than GOLD B patients. Indeed, a secondary analysis of the data of Koolen et al. [3] shows no differences in physical capacity and physical activity between GOLD B or D patients after stratification for patient profiles 2 to 5 (Fig. 2). Obviously, respiratory physiotherapy, including mucus evacuation techniques, needs to be offered if indicated [35].

**Table 2:** The number of reimbursed physiotherapy sessions in primary care for patients with profiles 3, 4 or 5 according to the proposed 2020 Dutch model

	Patient profile 3^a^	Patient profile 4^a^	Patient profile 5^a^
Pre-treatment screening sessions	2 days, 2 consecutive sessions/day	2 days, 2 consecutive sessions/day	2 days, 2 consecutive sessions/day
Number of weeks, treatment sessions per week	6 weeks, 2x/week 6 weeks, 1x/week	12 weeks, 3x/week	12 weeks, 3x/week
Intermediate evaluation sessions (12 weeks after start of therapy)	2 days, 2 consecutive sessions/day	2 days, 2 consecutive sessions/day	2 days, 2 consecutive sessions/day
Number of weeks, treatment sessions per week	18 weeks, 1x/2 weeks 12 weeks, 1x/4 weeks	14 weeks, 1x/week	14 weeks, 1x/week 18 weeks, 1x/2 weeks, 1x/4 weeks
Post-treatment evaluation session	2 days, 2 consecutive sessions/day	2 days, 2 consecutive sessions/day	2 days, 2 consecutive sessions/day
Total number of sessions	42	62	74

One session = 30 min

^a^We propose that GOLD stage 1 patients are also eligible for exercise-related care, as described Fig. 1; after a physician-treated COPD exacerbation, the profiling re-starts from the top of Fig. 1 and the number of sessions start from zero. Obviously, patients need to be encouraged to continue physical activity and/or training in and/or near their home-environment when the number of supervised physiotherapy sessions is decreasing

Understandably, the aims of the individual patient and the success of treatment should determine the total number of physiotherapy sessions. If the treatment in the primary care setting fails, referral for a thorough assessment of underlying pathophysiological mechanisms as well as for screening for interdisciplinary PR should be discussed with the patient and referral physician. Possible unknown, common medical comorbidities [36] may cause this failure, and should be actively screened for [37]. Moreover, the lack of motivation and the lack of social support, may also, at least partially, explain failure of therapy [38].

If the physiotherapy is successful (i.e., patient’s goals have been achieved), participation in regular sports/walking activities as organized for elderly in the local communities [17] seems feasible and should be recommended in the maintenance phase. Follow up of the physical capacity and physical activity of these patients by the physiotherapist over time (for example, two evaluative sessions every 6 months) seems sensible and should be considered for reimbursement. If the physical capacity has declined > 45 m on the 6-min walk test and/or the physical activity is > 1500 steps/day lower compared to previous assessment (i.e., a decline which exceeds 1.5 times the known MCID), a re-start of the physiotherapy should be deliberated in consultation with the treating physician. If a physician-treated exacerbation occurs during follow up, the profiling re-starts from the top of Fig. 1.

### High Disease Burden

Patients with COPD with a high self-reported disease burden (CCQ ≥ 1.9 points; CAT ≥ 18 points [24]), will be referred for a screening for PR. This is also proposed for patients who have recently been admitted to the hospital for an exacerbation, the so-called lung attack (Fig. 1; patient profile 6), as this is associated with significant deterioration of physical capacity, physical activity, and health status [39, 40]. Whether a patient should undergo a multidisciplinary PR program in secondary care, or a comprehensive, interdisciplinary, PR program in a Center of Expertise for Patients with Complex Chronic Lung Disease (tertiary care), depends on the degree of clinical complexity [18]. The clinical complexity may be operationalized by determining the number of treatable traits that is the number of potential targets for different rehabilitation ingredients [41, 42]. Obviously, also the interactions between treatable traits can also add to the clinical complexity, which is more challenging to operationalize [43]. As the degree of clinical complexity cannot be derived confidently from the degree of lung function impairment [4], a thorough pre-PR assessment has to determine the correct treatment allocation (Table 3). Patients with ≤ 2 treatable traits will be referred for an outpatient PR program in secondary care (outpatient, 8–12 weeks, three sessions per week, which will include physiotherapy and one or two other health disciplines, like health promotion and dietician; Fig. 1; patient profile 6A), while the remaining patients are appropriate candidates for a comprehensive, interdisciplinary PR program in a Centre of Expertise for Patients with Complex Chronic Lung Disease (specialized care; a minimum of 8 weeks, 3–5 PR days per week, with the possibility for inpatient stay), which will include physiotherapy, occupational therapy, dietary counseling, nutritional modulation, psychology, health promotion, enhanced art therapy, counseling by social work and respiratory nurse, respiratory medicine, and treatment of comorbidities (cardiology and internal medicine [18]; Fig. 1; patient profile 6B). Obviously, the inclusion criteria for treatment in a Centre of Expertise for Patients with Complex Chronic Lung Disease may change over time due to new insights. Indeed, cognitive functioning [44] and health literacy skills should be contemplated [45].

**Table 3:** Eligibility criteria for an interdisciplinary pulmonary rehabilitation program in a Centre of Expertise for Patients with Complex Chronic Lung Disease

Domain	Test	Criterion
Care dependency	Care Dependency Scale	≤ 68 points [51]
Body composition	Body weight	An unintentional loss in body weight of ≥ 5 kg in the last 12 months [52]
	Body mass index	< 18.5 kg/m^2^ or > 35 kg/m^2^
	Fat-free mass index	< 17 kg/m^2^ (men) or < 15 kg/m^2^ (women) [52]
Physical capacity	6-min walk distance	< 350 m [53]
	Incremental shuttle walk distance	70% predicted
Mobility and balance	Short Physical Performance Battery	≤ 9 points [54]
Symptoms of dyspnoea	Modified Medical Research Council	Grade ≥ 2 [55]
Symptoms of fatigue	Checklist Individual Strength—fatigue domain	≥ 36 points [56]
Symptoms of anxiety	Hospital Anxiety and Depression Scale	≥ 10 points on anxiety scale [57]
Symptoms of depression	Hospital Anxiety and Depression Scale	≥ 10 points on depression scale [57]
Adaptation to the disease burden	Nijmegen COPD Screening Instrument (NCSI)	Severe disease burden combined with ‘not adapted’ or ‘at risk’ [44]
Severe hypercapnia	Arterial blood gases	PaCO2 > 7.0 kPa
Cardiovascular comorbidities	Patient’s file	Under the care of a cardiologist
Exercise-induced oxygen desaturation despite providing O2-supplementation	Transcutaneous SpO2	< 90%

After completion of the PR program, including a structured outcome evaluation, the patients should be referred for at least once-weekly physiotherapy in the primary care setting to maintain the benefits in physical functioning and self-efficacy during the maintenance phase. To the future, E-health/M-health may also be put in place to coach and monitor patients during the enduring maintenance phase, which may stabilize physical capacity/activity levels, reduce in-person visits and may contribute to a better disease management [46, 47]. Robust evidence, however, is currently lacking [48].

## Discussion

In The Netherlands, about 600,000 people are diagnosed with COPD [19]. As the burden to the patients as well as to society is clearly present [49], the current organization of Dutch healthcare needs to make a transition towards an adequate referral of these patients to the different types of exercise-based care, including a healthy lifestyle advise, physiotherapy and/or PR. We propose the abovementioned patient profiling as a basis for this allocation. This type of matched care starts in the physician’s office or during an exacerbation-related hospital admission. Therefore, systematically quantifying the degree of disease burden and a subsequent referral, according to Fig. 1, should be considered as a future key process indicator. Clearly, large differences exist between countries concerning organizational aspects and content of exercise-based care programs for patients with COPD [50]. These local circumstances, as well as patient’s preference, may affect the proposed flowchart.

To date, use of primary care physiotherapy or PR is estimated to be very limited in The Netherlands (5.0 and 0.2%, respectively), while a larger proportion of the patients with COPD clearly qualify for this type of care [3]. So, improved referral rates are a necessity to lower the disease burden for patients and society. The proposed Dutch model has the intention to get the right patient allocated to the right type of exercise-related care and at the right moment, irrespective of the degree of airflow limitation. This will also avoid unnecessary exercise-related care expenses for patients with no-to-low disease burden and a preserved physical capacity/activity.
